# Concurrent Splenic Marginal Zone B Cell Lymphoma and Metastatic Pancreatic Adenocarcinoma Diagnosed on Splenectomy for Suspected Splenic Abscess

**DOI:** 10.7759/cureus.35541

**Published:** 2023-02-27

**Authors:** Kamilah Fernandez, Laraine H Cheung, Sabesan Balasinkam, Lekidelu Taddesse-Heath

**Affiliations:** 1 Pathology and Laboratory Medicine, Howard University Hospital, Washington, D.C., USA; 2 Pathology and Laboratory Medicine, Howard University College of Medicine, Washington, D.C., USA

**Keywords:** lymphoma, hematopathology and gastrointestinal pathology, ductal pancreatic adenocarcinoma, concurrent tumors, second primary malignancy, splenic cancer, multiple splenic abscess, incidental cancer, metastatic pancreatic adenocarcinoma, splenic marginal zone lymphoma

## Abstract

Splenic marginal zone lymphoma (SMZL) is an uncommon low-grade B-cell lymphoma. It is an indolent lymphoma with a median survival rate of greater than 10 years. Most patients are asymptomatic, but some patients may present with upper abdominal pain and distention, while others may present with splenomegaly, emaciation, fatigue, or weight loss. Due to the long median survival, patients with SMZL may develop a second primary malignancy.

Pancreatic adenocarcinoma is the most common malignant neoplasm of the pancreas. It has a poor prognosis with a five-year survival rate of 10%. Fifty percent of patients have metastatic disease on presentation. However, the spleen is not a common site of metastasis for malignant tumors from other primary sites including the pancreas. Here we present a case of a 78-year-old African American patient, who was found to have previously undiagnosed, concurrent metastatic pancreatic adenocarcinoma and SMZL diagnosed on splenectomy for a suspected splenic abscess.

## Introduction

Splenic marginal zone lymphoma (SMZL) is an uncommon, low-grade, B-cell non-Hodgkin lymphoma; however, it is the second most common subtype of marginal zone lymphoma, making up 20% of the cases [1]. This lymphoma is usually indolent, with approximately 10% converting to diffuse large B cell lymphoma [2]. Most patients are asymptomatic, presenting incidentally with splenomegaly and absence of lymphadenopathy. Some patients may present only with upper abdominal pain and distention, while others may present with splenomegaly, emaciation, fatigue, or weight loss [2].

In the United States, the median age at diagnosis for SMZL is 69 years [3], and the relative five-year overall survival rate is 85% [4]. Due to the long survival time of SMZL, patients may develop a second malignancy. Second primary cancers are observed with a higher incidence than expected in SMZL patients and include urinary tract and lung cancers [5].

The most common malignant neoplasm of the pancreas is pancreatic ductal adenocarcinoma [6]. Malignant exocrine pancreatic cancers are the leading cause of death for pancreatic neoplasms [7] and account for 8.2% of all cancer deaths reported in the United States [8]. Patients diagnosed with pancreatic adenocarcinoma have a five-year survival rate of 10% [9]. On initial presentation, more than 50% of patients have metastatic disease and metastasis will be detected in most patients who undergo resection [10]. The liver is the most common site of metastasis, followed by regional lymph nodes and the lung [11].

While a variety of hematologic malignancies commonly involve the spleen, secondary tumors of the spleen are rare since the spleen is not a common site of metastasis [12]. The most common sources of splenic metastases are melanoma and carcinomas from the breast, lung, colorectal region, and ovary [12]. Splenic metastases usually go unnoticed, but one case report has shown a rare presentation of splenic rupture due to metastasis presenting as acute abdomen in a patient with malignancy of the pancreas or biliary tract, both of which are rare primary sites for metastasis to the spleen [13]. Pancreatic carcinoma metastasis to the spleen is rare and occurs more frequently in association with tumors located in the body and tail of the pancreas.

While metastasis to the spleen is uncommon, the simultaneous occurrence of SMZL and metastatic carcinoma in the spleen is very rare. Here we present a case of a 78-year-old African American male with previously undiagnosed SMZL and metastatic pancreatic adenocarcinoma diagnosed on splenectomy for a suspected splenic abscess.

## Case presentation

**Figure 1:**
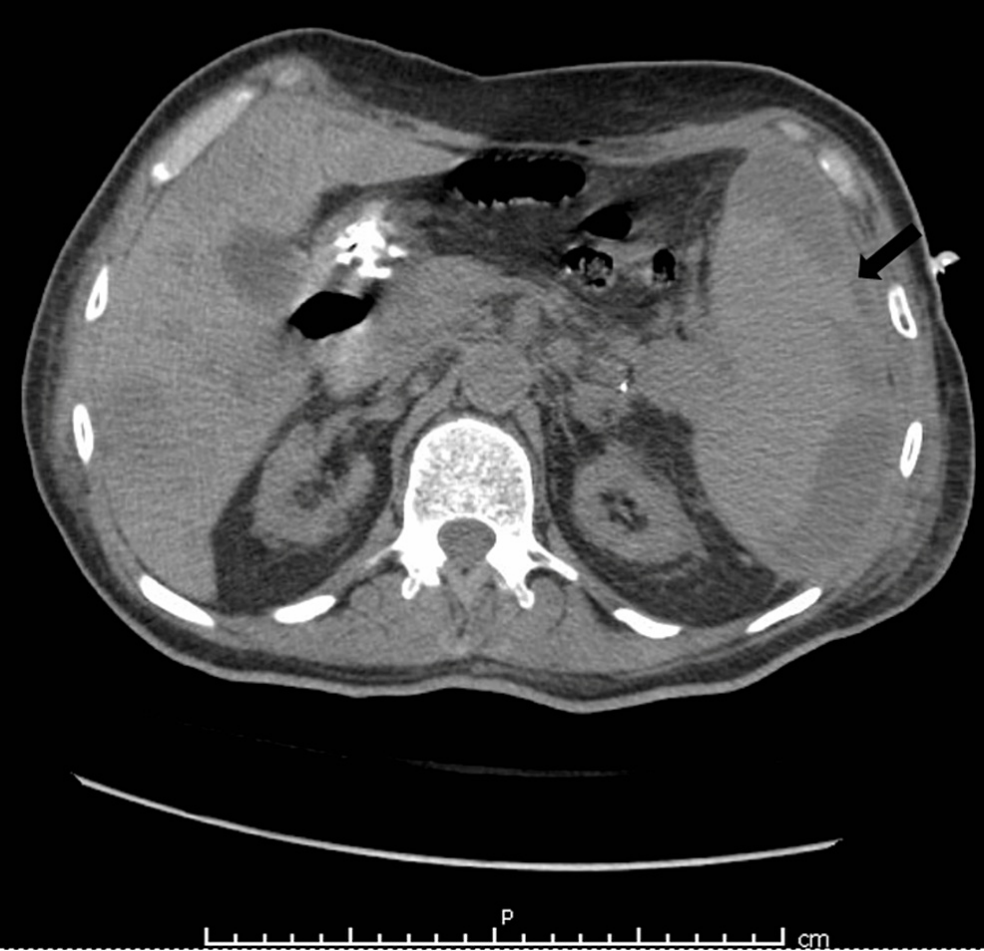
Computed tomography scan of the abdomen showing an enlarged spleen (arrow) with areas of hypoattenuation.

A 78-year-old male with a history of end-stage renal disease, hypertension, cerebrovascular accident, major depressive disorder, thrombocytopenia, anemia, and transient ischemic attack was brought to the emergency department from a nursing facility for altered mental status. On physical examination, the patient was non-communicative and disoriented and had a Glasgow Coma Scale (GCS) of 11/15. The patient’s vitals showed mild hypotension (BP 110/51 mmHg) but were otherwise unremarkable. Computed tomography (CT) scan of the head without contrast showed no intracranial hemorrhage or mass. Laboratory results were significant for elevated blood urea nitrogen/creatinine, low hemoglobin, elevated liver enzymes, and elevated troponins. CT of the abdomen and pelvis was subsequently done to evaluate hepatomegaly found on abdominal ultrasound and showed multiple well-demarcated low densities in the right lobes of the liver and a 1.8 cm low density in the left lobe of the liver with surrounding inflammatory hyperemia, suggestive of hepatic abscesses. There was marked splenomegaly measuring 15.1 cm in anteroposterior diameter and a 15 x 1.5 cm area of hypoattenuation involving the lateral periphery of the spleen and a possible suspicious lesion within the tail of the pancreas (Figure 1). Fine needle aspiration and biopsy of the left hepatic lobe were done which revealed infarction, but no malignancy was identified. After antibiotic treatment, a repeat CT of the abdomen and pelvis was performed which again showed splenomegaly with multiple hypoattenuated foci. The findings were suspicious for splenic abscess, infected splenic infarct, or infected splenic hematoma.

**Figure 2:**
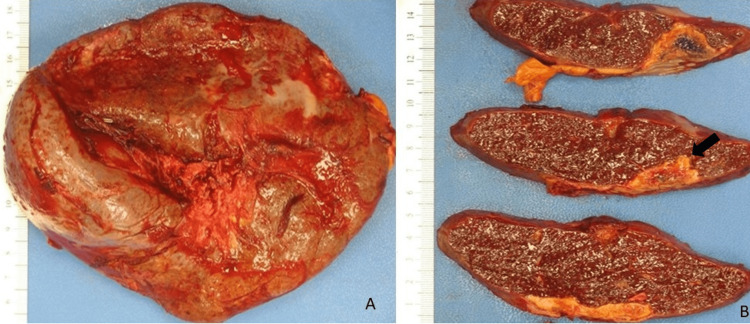
Gross images of the enlarged spleen. (A) The external surface is covered with purulent exudate. (B) Cut sections of the spleen showing well-demarcated areas of necrosis (arrow).

**Figure 3:**
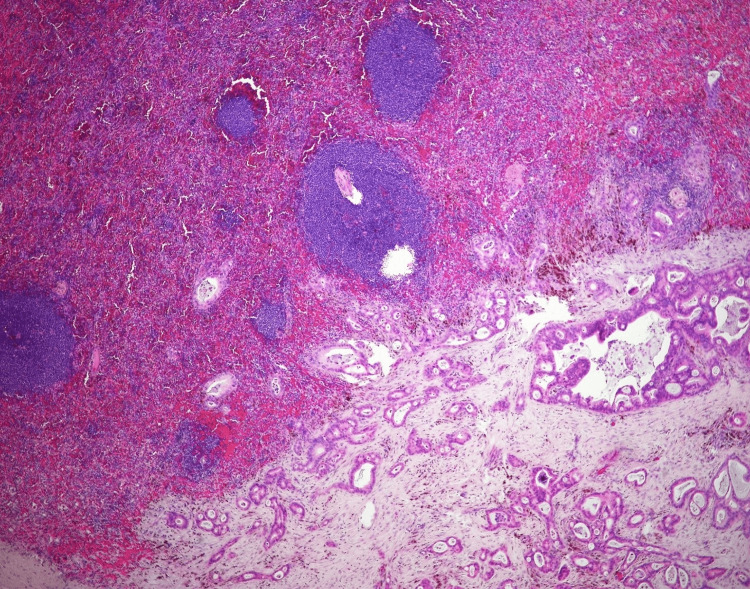
Microscopic sections of the spleen with metastatic carcinoma. The malignant glands infiltrate the splenic parenchyma with the marginal zone lymphoma.

**Figure 4:**
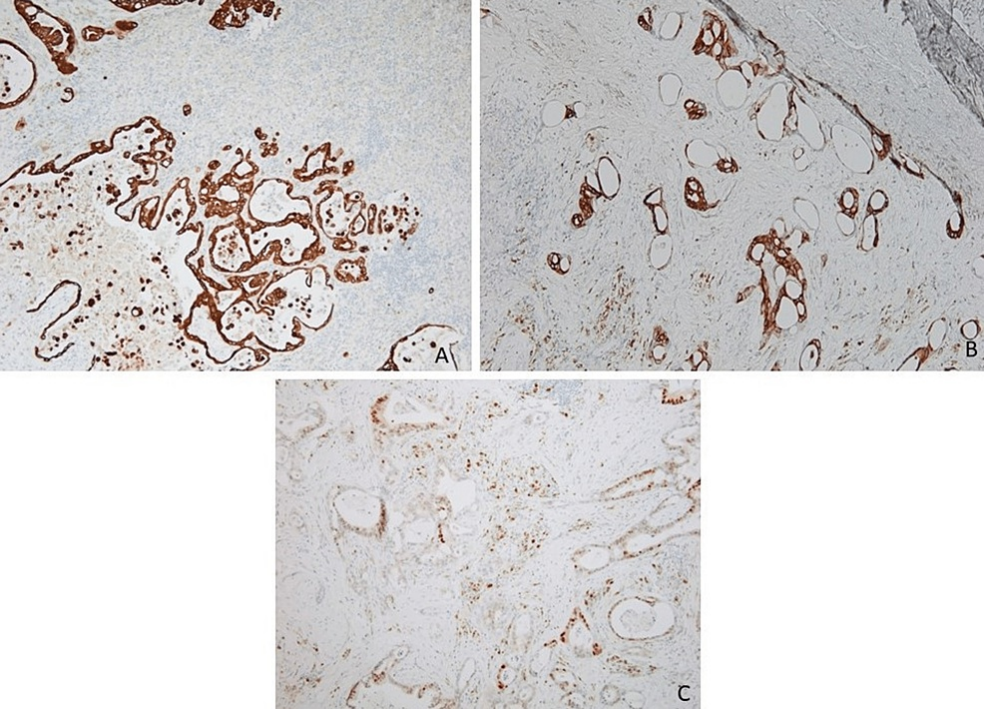
Immunohistochemistry stains of malignant glands. The tumor cells are strongly positive for CK7 (A) and show patchy positive staining for CK20 (B) and CDX2 (C).

**Figure 5:**
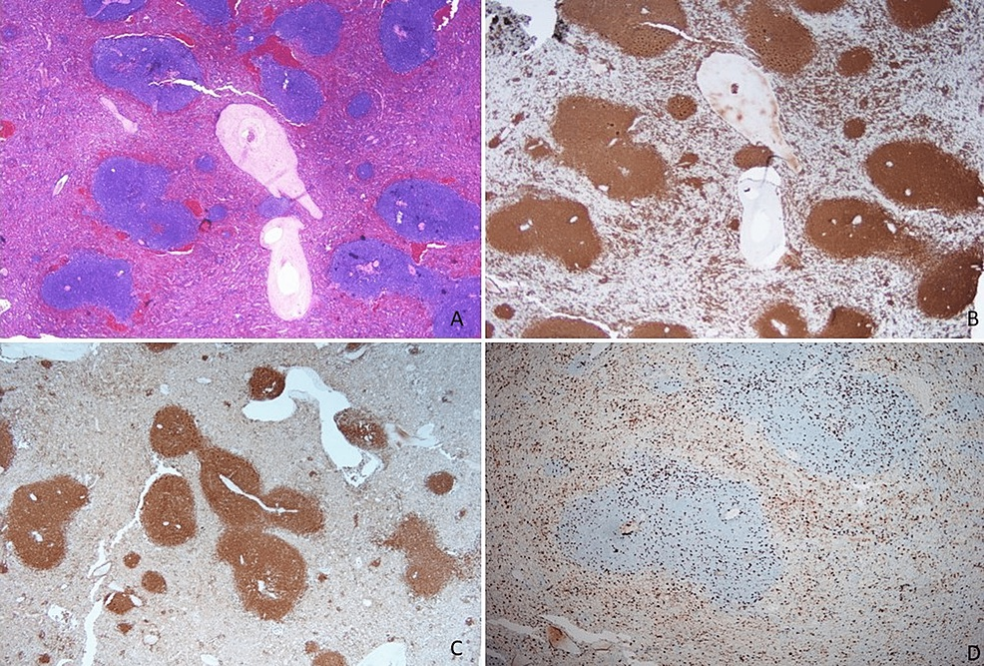
Microscopic sections of the spleen showing involvement by SMZL. There is expansion of the white pulp (A) by CD20 positive B-cells (B) that co-express BCL2 (C) and show a low proliferative rate with Ki-67 (D). SMZL: splenic marginal zone lymphoma

The patient underwent an exploratory laparotomy and splenectomy for multifocal splenic abscesses and source control for sepsis of indeterminate origin based on elevated lactate dehydrogenase (LDH), procalcitonin, and positive blood cultures for *Clostridium innocuum*. The spleen was examined by pathology. Grossly, the spleen measured 19 x 13 x 7 cm and weighed 587 grams. The external surface was covered with purulent exudate (Figure 2A). The cut surface revealed multiple well-demarcated areas of infarction and necrosis and foci of abscesses (Figure 2B). Histopathology revealed metastatic adenocarcinoma (Figure 3) associated with extensive/patchy areas of necrosis and immunostains showed strong diffuse CK7 positivity and patchy CK20 and CDX2 positivity (Figure 4), which was consistent with metastatic adenocarcinoma suggestive of pancreatic origin given the CT findings. The white pulp of the spleen was noted to be expanded with a proliferation of uniform small round lymphocytes, which were positive for CD20 and BCL2 and showed a low proliferation index for Ki-67 (Figure 5). The lymphocytes were negative for CD10, CD3, and CD5. Immunoglobulin heavy chain gene rearrangement performed by polymerase chain reaction revealed a clonal rearrangement. The morphologic, immunophenotypic, and clonality studies were consistent with splenic B-cell marginal zone lymphoma.

**Figure 6:**
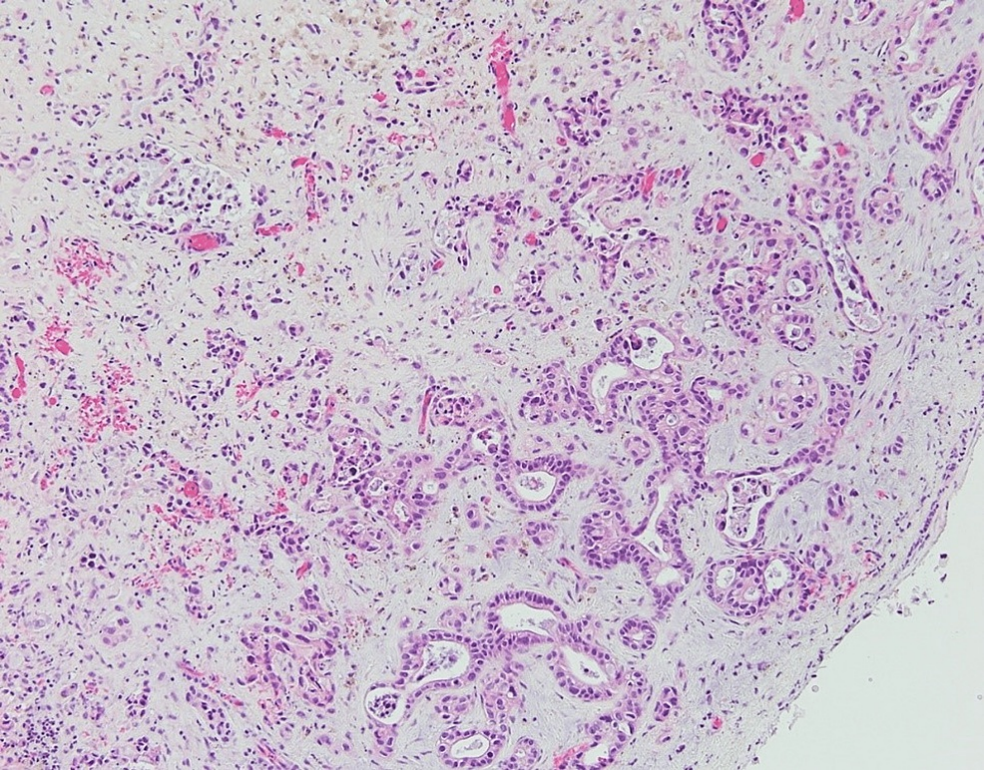
Microscopic image from liver biopsy showing infiltrating malignant glands surrounded by fibrosis.

**Figure 7:**
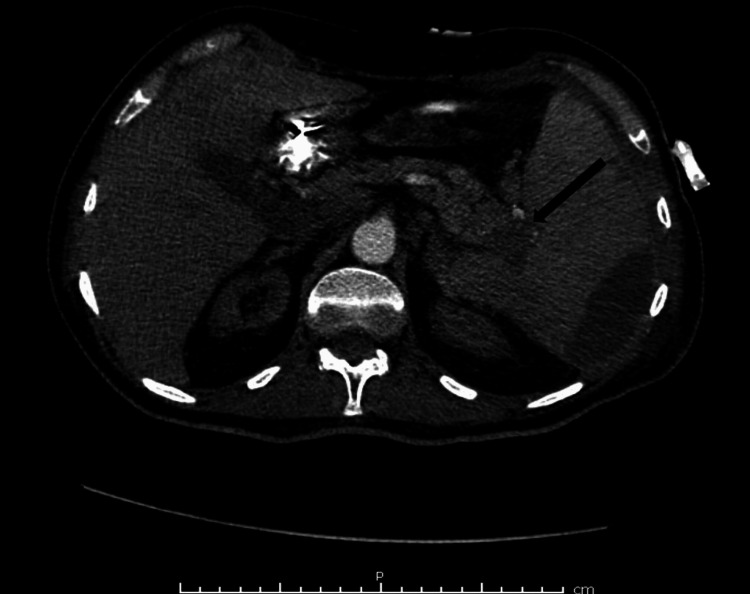
Repeat computed tomography scan of the abdomen showing hypoattenuated area in the tail of the pancreas (arrow).

A subsequent wedge biopsy of the liver was performed, and histopathology revealed metastatic adenocarcinoma (Figure 6) that was immunophenotypically identical to the metastatic adenocarcinoma in the spleen. A follow-up CT scan of the abdomen revealed a focal hypoattenuated area at the tail of the pancreas suspicious for pancreatic mass (Figure 7). The patient was given palliative treatment and discharged to hospice care but expired two weeks later.

## Discussion

Splenic marginal zone lymphoma is a rare neoplasm. It is a low-grade B cell lymphoma with a peak incidence occurring in the seventh decade of life, similar to the demographic of our 78-year-old patient. SMZL is a proliferation of small round lymphocytes in the splenic white pulp with an immunophenotypic profile of a B cell lymphoma positive for CD19, CD20, and BCL2, and a low Ki-67 proliferation index (Figure 5). Most patients are asymptomatic so the disease may not be diagnosed until significant symptoms and complications develop or the patient undergoes abdominal imaging studies as was the case with our patient. The multifocal necrosis and infarct associated with metastatic carcinoma and the patient's presentation led to a suspected diagnosis of splenic abscess, which was initially managed with antibiotic treatment; however, splenectomy was performed for source control of persistent sepsis, leading to histologic evaluation and accurate diagnosis.

Due to its indolent clinical course, with a median survival rate greater than 10 years, patients with SMZL may develop a second malignancy [5], and there have been numerous studies related to the long-term solid cancer risk among five-year survivors of both Hodgkin and non-Hodgkin lymphomas [14-16]. Our patient presented with metastatic pancreatic adenocarcinoma in the setting of an undiagnosed SMZL. Despite the uncertainty of how long our patient had SMZL, it is likely that the SMZL preceded the pancreatic adenocarcinoma since the patient previously had thrombocytopenia and anemia and SMZL is asymptomatic and indolent, whereas pancreatic adenocarcinoma follows a more aggressive course.

Pancreatic adenocarcinoma is 100% positive for CK7 [17]. CDX2 is a marker of gastrointestinal differentiation, and 33% of pancreatic adenocarcinoma can show weak and heterogeneous expression as seen in our patient [18]. CK20 was also expressed in our patient’s pancreatic adenocarcinoma and CK20 expression has been associated with distinct clinical features and predicts reduced survival [19].

Most pancreatic adenocarcinomas metastasize to the liver as is the case in our patient; however, splenic metastasis is very rare. A 10-year retrospective study by Ramanathan et al. of 135 patients with pancreatic adenocarcinoma showed that 16 patients (11.9%) had splenic involvement by pancreatic adenocarcinoma; 12 contiguous (direct extension) and four metastatic [20]. It is notable that most pancreatic carcinomas that metastasize to the spleen are located in the tail of the pancreas, which was also the case in our patient.

## Conclusions

In conclusion*, s*plenic metastasis is rare and more commonly found on autopsy. Our case demonstrates a very rare occurrence of a concurrent primary hematologic malignancy and metastatic adenocarcinoma in the spleen. Considering the radiographic overlap, this case further emphasizes the diagnostic and management challenges associated with malignancy-associated infarctions in the spleen occurring alongside splenic abscesses. Additionally, lymphoma patients need to be periodically followed long-term for the development of secondary primary malignancies, since they are reported to develop in some patients with lymphomas.
